# Eye-Tracking Metrics for Figure-Copying Processes in Early- vs. Late-Onset Alzheimer's Disease

**DOI:** 10.3389/fneur.2022.844341

**Published:** 2022-05-16

**Authors:** Ko Woon Kim, Jongdoo Choi, Juhee Chin, Byung Hwa Lee, Duk L. Na

**Affiliations:** ^1^Department of Neurology, Jeonbuk National University Medical School and Hospital, Jeonju, South Korea; ^2^Research Institute of Clinical Medicine of Jeonbuk National University, Jeonju, South Korea; ^3^Biomedical Institute of Jeonbuk National University Hospital, Jeonju, South Korea; ^4^Department of Neurology, Samsung Medical Center, Sungkyunkwan University School of Medicine, Seoul, South Korea; ^5^Neuroscience Center, Samsung Medical Center, Seoul, South Korea; ^6^Cell and Gene Therapy Institute (CGTI), Research Institute for Future Medicine, Samsung Medical Center, Seoul, South Korea; ^7^Alzheimer's Disease Convergence Research Center, Samsung Medical Center, Seoul, South Korea

**Keywords:** Alzheimer's disease, Rey–Osterrieth complex figure, eye-tracking, visuospatial dysfunction, copying process

## Abstract

Visuospatial dysfunction is a common symptom in patients with Alzheimer's disease (AD). To more focus on copying processes rather than on finally completed figures, we conceptually split the copying processes into three stages: visuoperceptual function, visuoconstructional function, and working memory function. We constructed perceptual and working spaces to investigate the different stages of copying, and then, we compared the number and duration of fixations and saccades and the number of switches across the two spaces. We used eye-tracking glasses to assess eye-tracking metrics in patients with early-onset AD (EOAD), patients with late-onset AD (LOAD), and normal control (NC) participants while they copied the simplified Rey–Osterrieth complex figure test (RCFT). Regarding eye metrics on the perceptual space, the number and duration of fixations were greater in both groups of patients with AD than in the NC participants group (number: EOAD vs. NC: *p* < 0.001, LOAD vs. NC: *p* = 0. 003/ duration: EOAD vs. NC: *p* < 0.001, LOAD vs. NC: *p* < 0.001). On the working space, the number and duration of fixations were greater in the patients with EOAD than in the patients with LOAD and NC participants (number: EOAD vs. LOAD: *p* = 0. 007, EOAD vs. NC: *p* = 0. 001/duration: EOAD vs. LOAD: *p* = 0. 008, EOAD vs. NC: *p* = 0. 002). The number of saccades and switching was higher in patients with EOAD than in NC participants (*p* < 0.001). The eye-tracking metrics from the simplified RCFT correlated with the neuropsychological test scores. Patients with EOAD and LOAD achieved the same level of performance at the simplified and original RCFT scores. However, patients with EOAD than LOAD showed a greater number and duration of fixations on the working space and more frequent switching between the perceptual and working spaces, which may reflect more cognitive efforts to achieve the same level of performance.

## Introduction

Visuospatial function is the ability to identify, integrate, and analyze space and visual form of objects and their spatial relations (1). Visuospatial dysfunction is a common symptom in patients with Alzheimer's disease (AD). This could be especially true in early-onset AD (EOAD), in which non-memory issues, including visuospatial processing, can be compromised as much as memory (2). Nevertheless, only a few studies have compared patients with EOAD and late-onset AD (LOAD) in terms of their visuospatial. functioning (3–7), and their results are inconsistent. A study showed that greater visuospatial impairment in patients with EOAD compared to patients with LOAD (6). Other studies showed that patients with EOAD had lower visuoconstructional function compared to patients with LOAD (4, 7). This inconsistency might stem from the test difficulty and AD severity or might indicate that the conventional scoring methods of visuospatial tests cannot show detailed differences.

The Rey–Osterrieth complex figure test (RCFT) is commonly used to assess patients' visuospatial behavior in patients with AD (8). The conventional scoring system of RCFT focuses on the shape and positional accuracy of the final drawings. However, individuals' drawing processes used to complete the figure have not been investigated fully, especially in patients with EOAD vs. LOAD. Previous studies suggest that the successful copying of figures such as RCFT requires neural or cognitive processes that include elementary visual function, visuospatial perception, basic motor and visuomotor coordination, attention/executive function, working memory, and planning and organizational abilities (9). However, after excluding factors such as elementary sensory/motor function and visuomotor coordination, we can narrow these processes down to three components. Normally, when people draw a complex figure, they look first at the target figure to perceive and hold the visual information and then draw the picture in the allocated space, going back and forth to complete the drawing. Therefore, as has been proposed in the previous studies (10, 11), first we conceptually split the copying processes into two essential components. The first component is when individuals are looking closely at the sample figure, which reflects the visuoperceptual function. The second component is when drawing or constructing the figure that the subject has seen in the perceptual space, which reflects the visuoconstructional function. Then, we added another component (the third component), which is when individuals are holding the information of the sample figure on the visuospatial sketchpad (12), reflecting the working memory function. Earlier studies also underscored the importance of visual memory or working memory when participants copy target figures (13, 14).

We posited that evaluation of copying processes would be more sensitive in detecting visuospatial abnormalities in patients with AD than would the conventional visual rating of RCFT that only scores shape and position of the completed figures. We also postulated that eye-tracking measurement would be useful in assessing the copying processes. Several studies have shown that eye-tracking measurement could be helpful for evaluating visual cognition (15–18). *Eye-tracking metrics* such as fixation, saccade, and smooth pursuit correlated with Mini-Mental State Examination (MMSE) scores (17), standard visuospatial test scores (16), and cortical thickness (15). However, previous studies used simple tasks such as saccade–antisaccade tasks, which are different from clinical settings where patients copy a target figure on a paper using a pen. Therefore, we requested participants draw a simplified version of RCFT in an experimental setting similar to a conventional neuropsychological test setting and obtained eye metrics related to the three stages of visual processing (visuoperceptual, visuoconstructive, and working memory). We hypothesized that the eye-tracking measurement in assessing the copying processes could serve as surrogate markers that help to detect early stages of AD. Despite inconsistencies, most studies suggest that visuospatial or visuoconstructive functions are more impaired in patients with EOAD than those with LOAD. Therefore, we further tried to fractionate drawing impairments by looking at whether patients with EOAD and LOAD differed in terms of the visuoperceptual, visuoconstructive, and working memory aspects of drawing.

## Materials and Methods

### Participant Recruitment

Participants were selected from those who visited the Memory Disorder Clinic at Samsung Medical Center in Seoul, Korea, between 1 June 2017 and 30 September 2018. This study group contained 19 EOAD, 19 LOAD, and 16 normal control (NC) participants. All patients with AD fulfilled the probable AD criteria proposed by the National Institute of Neurological and Communicative Disorders and Stroke and by the Alzheimer's Disease and Related Disorders Association (NINCDS-ADRDA) (19) and had MMSE score ≥ 10 and CDR ranging from 1 to 2 and therefore were diagnosed with mild to moderate AD. None of the patients with AD fulfilled the criteria for posterior cortical atrophy (PCA) (20, 21) or logopenic variant of primary progressive aphasia (22). Patients were divided into EOAD and LOAD groups based on the age of onset determined through an in-depth interview. Age of onset was defined as the first cognitive symptom based on an interview with close caregivers who saw the patient at least one time a week (23). We interviewed the caregiver about the first symptom of cognitive decline using the method proposed in the previous study (24). Patients with EOAD were defined as those whose first symptoms occurred between the ages of 45 and 65 years (25, 26). Patients with LOAD were defined as those whose first symptoms occurred after the age of 65 years (27). The NC participants were age-matched healthy controls aged 54–65 years, their scores on the neuropsychological battery were within one standard deviation of those of the age- and education-matched mean, and their MMSE score was 25 or higher (Table 1).

**Table 1:** Clinical information and cognitive profiles.

				***p*** **-values**		
	**EOAD (*n* = 19)**	**LOAD (*n* = 19)**	**NC (*n* = 16)**	**EOAD vs. LOAD**	**EOAD vs. NC**	**LOAD vs. NC**
**Age** (IQR)	64.5(63.0, 67.5)	78.0 (74.0, 79.0)	70.0(63.0, 73.0)	*<0.001*	*0*.318	*<0.001*
**Age of onset** (IQR)	61.0 (56.0, 64.0)	72.0 (69.0, 77.0)	N/A	*<0.001*	N/A	N/A
**Sex F:M**	12:7	12:7	7:9	>0.99	0.954	*0*.954
**Education** (IQR)	10.4 (8.0, 12.0)	12.0 (12.0, 16.0)	16.0 (12.0, 16.0)	0.645	0*.009*	*0*.288
**APOE4 carrier** ^**a**^	12/18 (66.7%)	8/19 (42.1%)	0/12 (0%)	0.574	0*.001*	*0.035*
**Amyloid PET positive** ^**b**^	17/17	7/7	0/4	N/A^c^	N/A^c^	N/A^c^
**Attention**						
Forward digit span (IQR)	6 (4, 7)	6 (5, 7)	7 (6, 8)	>0.99	0.058	*0*.033
Backward digit span (IQR)	3 (2, 4)	3 (2, 4)	5 (4, 7)	>0.99	0*.001*	*0.003*
**Language**						
K-BNT (IQR)	44 (22, 48)	29 (16, 37)	54 (49, 57)	*0.141*	*0.002*	*<0.001*
Calculation (IQR)	9 (8, 12)	9 (7, 12)	12 (12, 12)	>0.99	*0.004*	*0.002*
**Visuospatial function**						
RCFT: copying (IQR)	28.0 (12.5, 32.0)	25.0 (4.5, 29.0)	35.0 (34.0, 35.5)	0.966	*0.001*	*<001*
**Memory**						
SVLT: immediate recall (IQR)	12 (7, 14)	9 (8, 11)	24 (21, 26)	0.812	*<0.001*	*<0.001*
SVLT: delayed recall (IQR)	0 (0, 0)	0 (0, 0)	8 (7, 10)	>0.99	*<0.001*	*<0.001*
SVLT: recognition (IQR)	15 (12, 17)	15 (14, 16)	23 (22, 24)	>0.99	*<0.001*	*<0.001*
RCFT: immediate recall (IQR)	2.5 (1.0, 3.0)	0.5 (0.5, 3.0)	21.5 (15.5, 28.0)	>0.99	*<0.001*	*<0.001*
RCFT: delayed recall (IQR)	0.0 (0.0, 0.0)	0.0 (0.0, 2.5)	20.0 (15.5, 27.0)	>0.99	*<0.001*	*<0.001*
RCFT: recognition (IQR)	16.0 (14.0, 17.0)	15.0 (14.0, 17.5)	21.5 (20.0, 22.0)	>0.99	*<0.001*	*<0.001*
**Frontal/executive functions**						
COWAT animal (IQR)	10 (6, 12)	9 (5, 11))	19 (17, 23)	>0.99	*<0.001*	*<0.001*
COWAT supermarket (IQR)	9, (8, 10)	9 (7, 11)	19 (15, 23)	>0.99	*<0.001*	*<0.001*
COWAT phonemic (IQR)	13 (11, 20)	11 (7, 19)	37 (33, 45)	>0.99	*<0.001*	*<0.001*
Stroop test: color (IQR)	46 (19, 71)	25 (5, 61)	111 (86, 112)	>0.99	*<0.001*	*<0.001*
**MMSE** (IQR)	19 (16, 24)	18 (16, 20)	30 (29, 30)	>0.99	*<0.001*	*<0.001*
**CDR** (IQR)	1.0 (1.0, 1.5)	1.0 (1.0, 2.0)	0.5 (0.5, 0.5)	0.946	*<0.001*	*<0.001*
**CDR sum of box** (IQR)	5.5 (5.0, 9.5)	7.0 (5.0, 10.0)	0.5 (0.5, 0.5)	>0.99	*<0.001*	*<0.001*
**GDS** (IQR)	1.5 (1.0, 3.5)	3.5 (1.0, 7.0)	1.5 (0.0, 6.0)	>0.99	*<0.001*	*<0.001*

*Data are presented as the median and IQR (interquartile range) for continuous variables, and the Kruskal–Wallis test and post hoc assessments with Dunn's pairwise tests were used for comparisons*.

*chi-square test was used to compare categorical variables*.

*APOE4, apolipoprotein E4; EOAD, early-onset Alzheimer's disease; CDR, Clinical Dementia Rating; COWAT, Controlled Oral Word Association Test; K-BNT, Korean version of the Boston Naming Test; LOAD, late-onset Alzheimer's disease; MMSE, Mini-Mental State Examination; N/A: not accessible; NC, normal control; PET, positron emission tomography; RCFT, Rey–Osterrieth complex figure test; SVLT, Seoul Verbal Learning Test*.

a *APOE4 was analyzed in 49 patients: 18 patients with EOAD, 19 patients with LOAD, and 12 NCs. Participants with one or more copies of the ε4 allele (i.e., ε2/4, ε3/4, ε4/4) were considered to be ε4 carriers (28)*.

b *Amyloid PET was analyzed in 28 patients: 17 patients with EOAD, 7 patients with LOAD, and 4 NCs. Amyloid PET positivity was interpreted based on the previously reported guidelines for each ligand (29, 30)*.

c *Statistical analysis of amyloid PET positivity between EOAD vs. LOAD, EOAD vs. NC, and LOAD vs. NC groups were not available because all patients with AD tested positive, and all NC participants tested negative in their amyloid scans*.

*The italic values show significant differences at P values < 0.05*.

We consecutively recruited participants who satisfied the following criteria: (i) normal or corrected-to-normal visual acuity, (ii) more than 6 years of education, (iii) completion of a standardized neuropsychological battery: the Seoul Neuropsychological Screening Battery (SNSB) (31, 32), the Mini-Mental State Examination (MMSE) test (33), and (iv) magnetic resonance imaging (MRI). Participants were evaluated *via* extensive diagnostic procedures, including detailed clinical interviews and neuropsychological testing, blood tests, imaging (MRI and positron emission tomography [PET] scans), and a clinical consensus of neurologists, neuropsychologists, and radiologists. We excluded participants with diseases that could affect cognitive function. Individuals with moderate or severe vision loss (visual acuity <0.3) or a very low MMSE score (lower cutoff at 10) or an education level lower than the 6th grade were excluded from this study. All experimental procedures were performed in compliance with the protocols approved by the Institutional Review Board of Samsung Medical Center, and written informed consent was obtained from all the participants.

### Neuropsychological Assessments

All participants underwent a standardized neuropsychological battery called the SNSB (31, 32), which consists of the following tests. Attention and working memory were assessed with the forward and backward digit span tests (34); language was tested using the Korean version of the Boston naming test (K-BNT) (35); calculation was tested with three items each for addition, subtraction, multiplication, and division; visuospatial function was assessed with the Rey–Osterrieth complex figure test (RCFT) (8); memory function was assessed using immediate and delayed recall of the Seoul Verbal Learning Test (SVLT) (36) and RCFT; and frontal-executive function was tested with the phonemic and semantic Controlled Oral Word Association Test (COWAT) (37) and the Stroop word/color reading test (38). Participants also were tested with the MMSE, Clinical Dementia Rating (CDR) and CDR sum of box, and Geriatric Depression Scale (GDS) for assessing general cognition and depression.

### Experimental Apparatus

We used SMI Eye-Tracking Glasses 2 Wireless (SMI ETG 2w, SensoMotoric Instruments, Germany), which record binocular eye movements at a 120 Hz sampling rate and a 1,280 x 960 pixel resolution scene camera. The eye tracker has a reported gaze estimation accuracy of 0.5° and precision of 0.1°. The experimental equipment to measure the figure-copying performance consisted of a digital tablet (size = 12 inches, resolution = 2,160 x 1,440, Samsung Galaxy Book 12, Samsung Electronics, Suwon, Korea) and a digital pen (nib diameter = 0.7 mm, pressure = 4,096, S-pen, Samsung Electronics, Suwon, Republic of Korea).

### Experimental Design

#### Calibration

Participants were instructed to wear the eye-tracking glasses and look at a static calibration point in the middle of the tablet for 5 s. While the participant was looking at the point, the experimenter adjusted the participant's focus to the center of the tablet using the recording computer. After calibration, points appeared at several random positions on the tablet for 1 s each, and we tested whether the participant's gaze followed them well. The experiment was started when the participants performed the procedure well; otherwise, calibration was repeated whenever necessary.

#### Simplified RCFT Copying

**Figure 1:**
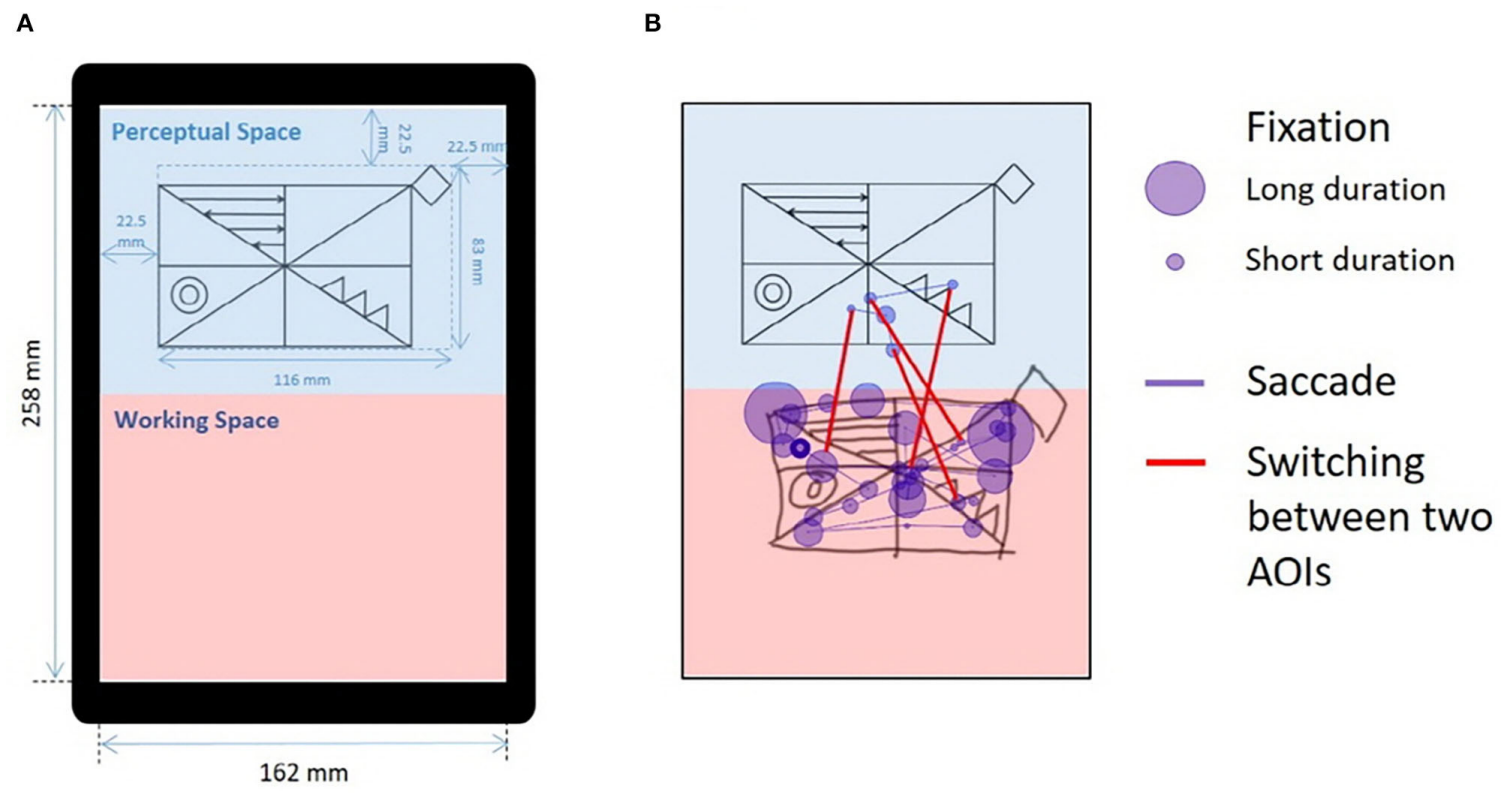
Drawing from the simplified RCFT and an illustration of eye-tracking metrics. **(A)** Modified figure from previous publication (39). The simplified RCFT was shown on a tablet computer (size = 12 in, resolution = 2,160 x 1,440, Samsung Galaxy Book 12, Samsung Electronics, Suwon, Korea) with a screen width of 162 mm and a screen height of 258 mm. The upper half was defined as the “perceptual space,” and the lower half was defined as the “working space.” The simplified Rey figure presented in the perceptual space was 152 mm in width and 95 mm in height. The left, right, and upper margins were 30 mm. The blue text, markers, and background colors were not presented to the participants. **(B)** An illustration of the eye movements made by a normal individual while drawing the simplified RCFT for 15 s. We measured three indices from the eye movements: (1) fixations, (2) saccades, and (3) switches between the two AOIs. The purple circles indicate fixations, and the size of each circle indicates the duration of the fixation. The purple lines connecting the fixations indicate saccades. The red lines across the blue perceptual space and red working space indicate switches between the two spaces. AOI, area of interest; RCFT, Rey–Osterrieth complex figure test.

In previous research, we modified the original RCFT into a simpler version and validated our simplified RCFT against the original RCFT (39). Our simplified RCFT comprises 4 global (large rectangle and diagonal, horizontal, and vertical crosses) and 4 local (a square, double circles, three triangles, and four arrows) components (Figure 1A). We scored our simplified RCFT in terms of both accuracy and placement, and our scoring complied with the Meyers and Meyers' standardized scoring of the original RCFT. Raw scores ranged from 0.0 to 16.0.

The participants in this study performed the simplified RCFT while wearing the eye-tracking glasses. We defined the upper half of the portrait-oriented screen as the “perceptual space” and the lower half as the “working space” (Figure 1A). The sample figure of the simplified RCFT was presented in the perceptual space. We postulated that fixations or saccades in perceptual space may reflect a visuoperceptual component; fixations or saccades in working space may reflect a visuoconstructional component; switches (saccades) between the two spaces may reflect a working memory component.

Prior to the task, the experimenter delivered the following instructions: “*A sample figure will appear in the upper half of this tablet when the task starts. Please copy the figure with this pen. Try to use the empty area below as much as possible. This pen does not have an eraser. You cannot change your drawing. If you make a mistake, forget about it and keep going with the rest. Take your time, and let me know when you are done.”* The completion time of this simplified RCFT was defined as the time interval from the moment immediately after the oral instruction was given to the moment when the participant announced that they had completed the drawing.

The pen the participants used was a digital pen for pen trajectory data acquisition. The digital pen data from a subset of the individuals who participated in this study were previously published elsewhere (39).

### Measurement of Eye-Tracking Metrics

We used SMI BeGaze version 3.4 analysis software to map gaze data from the scene videos, which is called semantic gaze mapping, and to quantify multiple participant data (40). As illustrated in Figure 1B, we measured three metrics from participants' eye movements for the two areas of interest (AOIs), the perceptual space and the working space: (1) number and duration of fixations in the perceptual and working spaces, (2) number and duration of saccades in the perceptual and working spaces, and (3) switches between the perceptual space and the working space.

*1. Number and duration of fixations*: Fixations were defined as groups of consecutive points within a particular dispersion. The minimum fixation duration was 80 ms and the maximum dispersion value was 100px (41). We measured the number and average duration of fixations on the two AOIs. We also calculated the ratio of the number and duration of fixations between the two AOIs (working space/perceptual space).

*2. Number and duration of saccades*: Saccades were defined as fast eye movements that occurred when participants shifted their gaze from one target to the other. A peak is defined as the peak value of velocities above the peak threshold of 40°/s. The peak could indicate a saccade if the distance between the start and end exceeds the minimum saccade duration of 22 ms and the single peak value lies in the range of 20–80% of the distance between the start and end (41). We measured the number and average duration of saccades.

*3. Switching between two AOIs*: Switches were defined as saccades made from one AOI to the other. We measured the number of switches between the two AOIs.

### Statistical Analysis

To validate the simplified Rey figures, we used the Pearson correlation to compare conventional RCFT scores with those of the simplified RCFT. To determine consistency among raters, we calculated an intraclass correlation coefficient (ICC). A two-way random-effect model based on single rating and absolute agreement assessed the inter-rater repeatability. We used the Kolmogorov–Smirnov test to verify the normality of the demographics, cognitive profile data, and eye-tracking metrics. We used the Kruskal–Wallis (KW) test to examine statistical differences between groups at a significance level of *p* = 0.05 because the variables did not follow a normal distribution. We performed *post hoc* comparisons using Dunn's pairwise tests. We used the chi-square test or Fisher's exact test for categorical variables followed by Bonferroni *post hoc* analysis. To test whether the eye-tracking metrics correlated with age or education, we used Pearson's correlation. We performed multiple linear regression to provide a way of adjusting for the completion time. To test correlations between the eye-tracking metrics and neuropsychological test scores in combined EOAD and LOAD groups, we used Pearson's correlation. We performed all statistical analyses using SPSS version 24.0 for Windows.

## Results

### Clinical Information and Cognitive Profiles

This study group comprised 19 patients with EOAD, 19 patients with LOAD, and 16 NCs. Among them, 18 of 19 EOAD, 7 of 19 LOAD, and 4 of 16 NC underwent either florbetaben (19/29) or flutemetamol (10/29) amyloid PET. Amyloid PET positivity was interpreted according to the guidelines of each PET ligand (20, 29). All patients with AD tested positive, and all NC participants tested negative in their amyloid scans. Age (*p* < 0.001) and education (*p* = 0. 012) differed among the EOAD, LOAD, and NC participants. Patients with EOAD and LOAD showed the poorer performance in attention, language function, visuospatial function, memory, and frontal/executive function testing than the NC participants. There were no significant differences between EOAD and LOAD in dementia severity and level of depression. The prevalence of *APOE4* carriers among the NC participants (0%, 0/12) was significantly lower than that among the patients with EOAD (66.7%, 12/18) and LOAD (42.1%, 8/19) (Table 1).

### Validity and Inter-rater Reliability of the Simplified Rey–Osterrieth Complex Figure Test

The copying results from the simplified RCFT were scored separately by three raters (two neuropsychologists and one neurologist). The ICC for inter-rater reliability was excellent, 0.99 (95% CI: 97–0.99, *p* < 0.001). All participants completed both simplified and original RCFTs. The average scores (mean ± standard deviation) of the three raters on the simplified and original RCFT scores were 9.4 ± 4.7 and 20.0 ± 12.4 in patients with EOAD, 10.4 ± 5.2 and 21.8 ± 13.2 in patients with LOAD, and 14.6 ± 1.1 and 34.2 ± 1.7 in NC participants, respectively. The groups differed significantly (Kruskal–Wallis test, *p* < 0.001), and the *post hoc* test showed that the visual rating scores in both the simplified and original RCFT were lower in both groups of patients with AD than among the NC participants (Dunn's pairwise tests, EOAD vs. NC: *p* < 0.001 and *p* < 0.001, LOAD vs. NC: *p* = 0. 002 and *p* < 0.001,). However, the performances of the patients with EOAD and LOAD did not differ from each other. There was a linear relationship between the average scores of the three raters on the simplified RCFT and original RCFT scores (*r* = 0.792; *p* < 0.001).

### Eye-Tracking Metrics

Before analyzing the eye-tracking data, we reviewed it carefully and excluded the data that were not appropriate. A number of four participants were excluded because of program input error. A number of one participant was excluded because of visual disturbance. Therefore, the eye-tracking metrics were analyzed from a total of 17 patients with EOAD, 16 patients with LOAD, and 16 NC participants. None of the eye-tracking metrics showed any significant correlation with age or education. After data collection and gaze mapping were completed, graphical representations of the eye movements over the scene were developed for further analysis. The eye-gaze patterns of all participants were mapped onto three information-format images. Table 2 shows the statistical analysis results for the eye-tracking metrics and the completion times. Patients with EOAD and LOAD showed longer completion time than the NC participants, but there was no significant difference between the EOAD and LOAD groups (Table 2).

**Table 2:** Eye-tracking metrics.

				***p*** **-values**					
	**EOAD (*n* = 17)**	**LOAD (*n* = 16)**	**NC (*n* = 16)**	**EOAD vs. LOAD**		**EOAD vs. NC**		**LOAD vs. NC**	
				***r***	***P***	***r***	***p***	***r***	***p***
***Number of fixations***									
Total number of fixations	282 (163, 455)	182 (120, 250)	118 (98, 146)	0.314	0.219	0.724	*<0.001*	0.420	0.053
Number of fixations-perceptual AOI	118 (60, 187)	67 (50, 121)	34 (25, 46)	0.194	>0.99	0.721	*<0.001*	0.613	0*.003*
Number of fixations-working AOI	164 (113, 218)	86 (58, 136)	81 (69, 105)	0.474	0*.007*	0.687	*.001*	0.047	>0.99
Ratio (working/perceptual AOI)	1.47 (1.07, 1.68)	1.34 (0.47, 2.07)	2.40 (1.71, 3.59)	0.125	>0.99	0.608	*.005*	0.533	*.002*
***Fixation duration (s)***									
Total fixation duration	105.18 (62.43, 157.40)	60.07 (43.16, 74.44)	41.84 (30.38, 52.35)	0.357	0.185	0.721	*<0.001*	0.466	0*.043*
Fixation duration-perceptual AOI	30.50 (18.13, 56.57)	17.99 (12.13, 43.48)	7.22 (5.07, 10.09)	0.176	>0.99	0.809	*<0.001*	0.720	*<0.001*
Fixation duration- working AOI	54.85 (43.88, 79.37)	31.99 (20.97, 50.17)	34.58 (22.13, 42.98)	0.489	0*.008*	0.615	*.002*	0.033	>0.99
Ratio (working/perceptual)	2.06 (1.37, 2.64)	1.71 (0.46, 3.21)	4.84 (3.08, 7.92)	0.113	>0.99	0.671	*.001*	0.633	*<0.001*
***Saccades***									
Total number of saccades	267 (154, 397)	169 (99, 238)	105 (85, 137)	0.298	0.200	0.712	*<0.001*	0.353	0.100
Total duration of saccades (s)	4.34 (3.75, 4.40)	3.99 (3.83, 4.46)	4.32 (3.82, 4.76)	0.016	>0.99	0.125	>0.99	0.187	>0.99
***Switches***									
Number of switches	80 (62, 107)	50 (31, 83)	36 (30, 47)	0.373	0*.049*	0.772	*<0.001*	0.267	0.227
***Completion time of simplified RCFT (s)***	110.88 (65.09, 167.06)	64.81 (47. 53, 79.09)	45.43 (35.28, 58.18)	0.376	0.152	0.727	*<0.001*	0.466	0*.046*

*Data are presented as the median and IQR (interquartile range) for continuous variables, and the Kruskal–Wallis test and post hoc assessments with Dunn's pairwise tests were used for comparisons. Effect size (r) was calculated with the following formula $r=\frac{\left|z\;\right|}{\sqrt{n}}$*.

*AOI, area of interest; EOAD, early-onset Alzheimer's disease; LOAD, late-onset Alzheimer's disease; NC, normal control*.

*The italic values show significant differences at P values < 0.05*.

#### Number and Duration of Fixations

There was an overall trend observed that the patients with EOAD showed a greater number and duration of fixations compared with the patients with LOAD. The patients with LOAD showed a similar trend compared with NC participants. However, statistical analyses only showed that the total number of fixations was higher in the patients with EOAD than among the NC participants (Dunn's pairwise tests, *p* < 0.001,). Patients with EOAD and LOAD did not differ in terms of total fixation number. The total fixations duration was longer in both patient with AD groups than among the NC participants (Dunn's pairwise tests, EOAD vs. NC: *p* < 0.001, LOAD vs. NC: *p* = 0.043).

**Figure 2:**
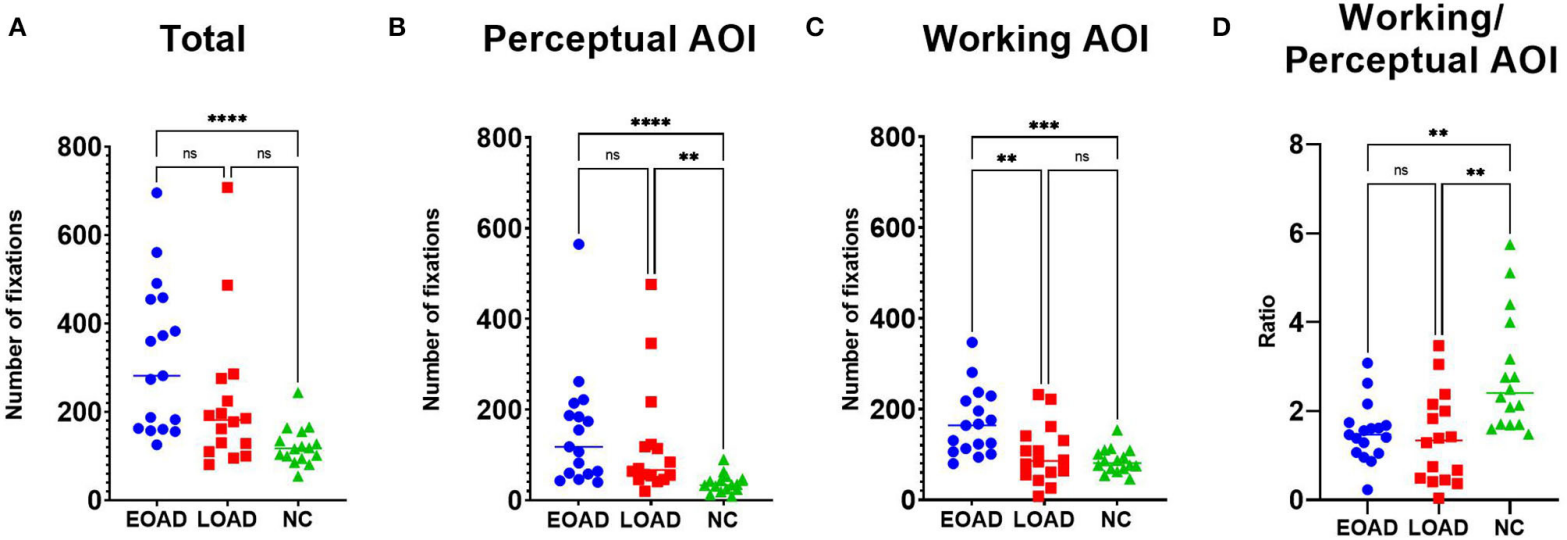
The number of fixations. **(A)** Patients with EOAD showed more frequent fixations than NC participants. **(B)** Patients with EOAD and LOAD showed more frequent fixations on the perceptual space AOI than the NC participants. **(C)** Patients with EOAD showed more frequent fixations on the working space AOI than the LOAD and NC participants. **(D)** Patients with EOAD and LOAD showed a higher ratio (working/perceptual space) of fixations on the AOIs than the NC participants. AOI, area of interest; EOAD, early-onset Alzheimer's disease; LOAD, late-onset Alzheimer's disease; NC, normal control. *p < 0.05, **p < 0.01, ***p < 0.001, ****p < 0.0001.

The median total number of fixations was 282 in patients with EOAD, 182 in patients with LOAD, and 118 in NC participants (Figure 2A). The number of fixations on the perceptual AOI was higher in the AD groups than in the NC group (Dunn's pairwise tests, EOAD vs. NC: *p* < 0.001, LOAD vs. NC: *p* = 0. 003) (Figure 2B). The number of fixations on the working AOI was higher in the patients with EOAD than in the patients with LOAD and NC participants (Dunn's pairwise tests, EOAD vs. LOAD: *p* = 0. 007, EOAD vs. NC: *p* = 0. 001) (Figure 2C). The ratio of the number of fixations between the two AOIs (working space/perceptual space) was lower in the patients with AD than in the NC participants (Dunn's pairwise tests, EOAD vs. NC: *p* = 0. 005, LOAD vs. NC: *p* = 0. 002) (Figure 2D).

**Figure 3:**
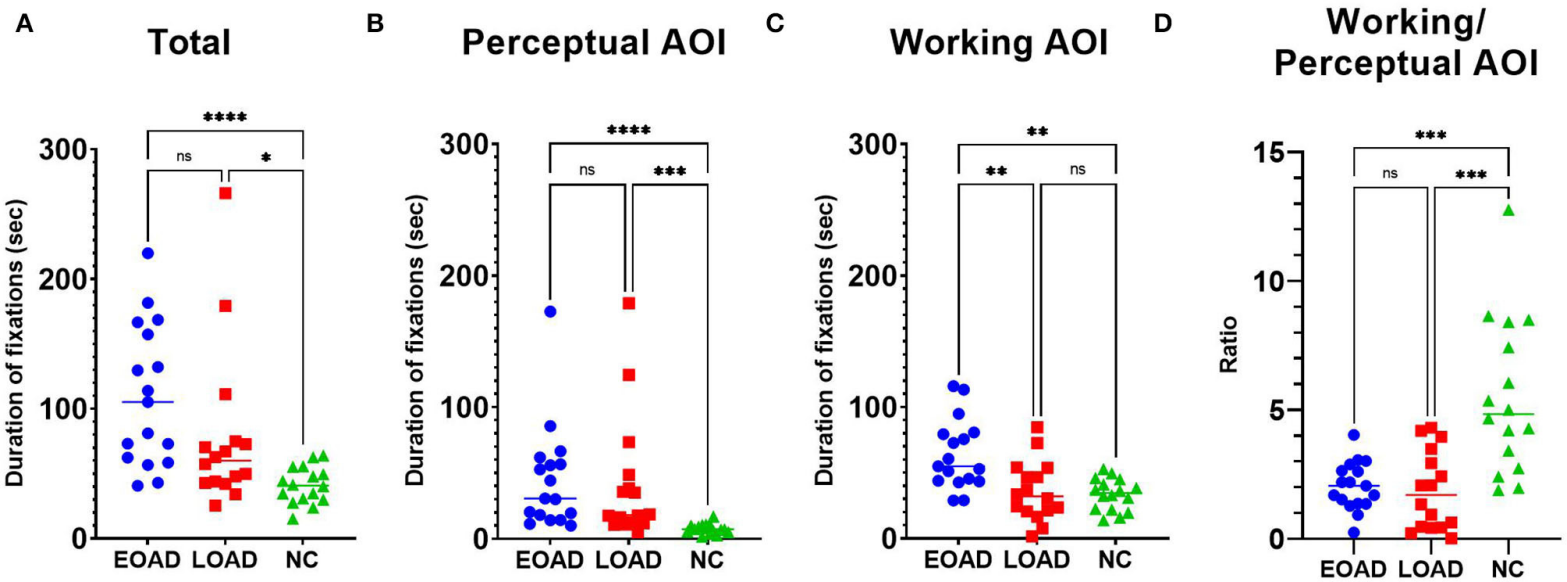
The fixation duration. **(A)** The total fixations duration was longer in both patient groups with AD than among the NC participants. **(B)** Patients with EOAD and LOAD showed longer fixation durations on the perceptual space AOI than NC participants. **(C)** Patients with EOAD showed longer fixation durations on the working space AOI than LOAD and NC participants. **(D)** Patients with EOAD and LOAD showed a lower ratio (working/perceptual space) of fixation durations on the AOIs than NC participants. AOI, area of interest; EOAD, early-onset Alzheimer's disease; LOAD, late-onset Alzheimer's disease; NC, normal control. *p < 0.05, **p < 0.01, ***p < 0.001, ****p < 0.0001.

The median total fixation duration was 105 s in patients with EOAD, 60 s in patients with LOAD, and 41 s in NC participants (Figure 3A). The fixation duration on the perceptual space AOI was longer among patients with AD than NC participants (Dunn's pairwise tests, EOAD vs. NC: *p* < 0.001, LOAD vs. NC: *p* < 0.001) (Figure 3B). The fixation duration on the working space AOI was longer among patients with EOAD than patients with LOAD and NC participants (Dunn's pairwise tests, EOAD vs. LOAD: *p* = 0. 008, EOAD vs. NC: *p* = 0. 002) (Figure 3C). The ratio of the fixation duration between the two AOIs (working space/perceptual space) was lower in both AD groups than in the NC group (Dunn's pairwise tests, EOAD vs. NC: *p* = 0. 001, LOAD vs. NC: *p* < 0.001) (Figure 3D).

Multiple linear regression analyses with completion time (Table 2) as a covariate showed that after adjusting for completion time, only the ratio of number (working/perceptual AOI) (*p* < 0.001) and the ratio of duration (working/perceptual AOI) (*p* < 0.001) measurements were associated with both AD vs. NC group differences. Multiple linear regression analyses with completion time (Table 2) as a covariate showed that number (*p* = 0. 026) and duration (*p* = 0. 008) of fixations on the working AOI were associated with EOAD vs. LOAD group differences.

#### Number and Duration of Saccades

**Figure 4:**
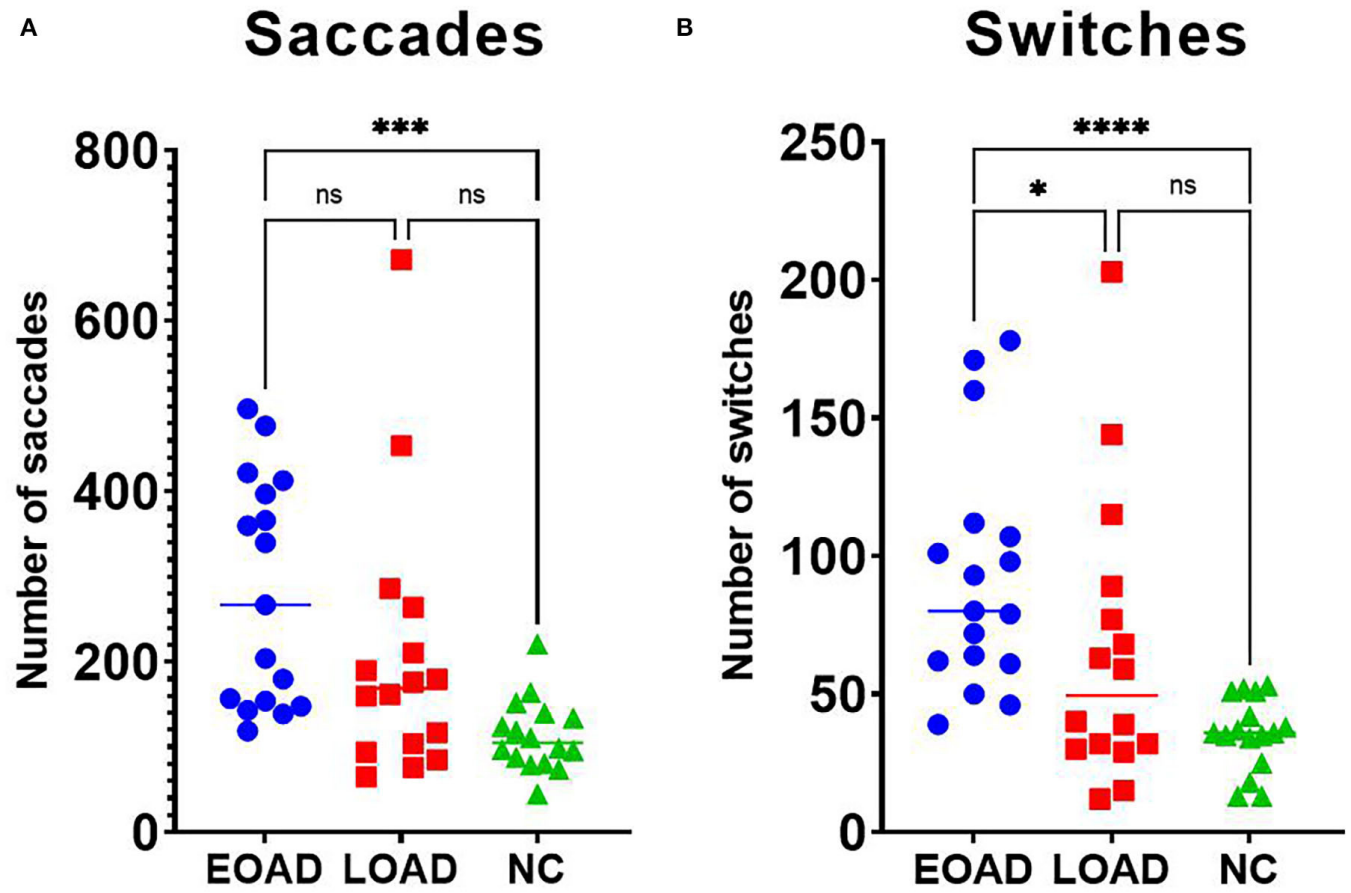
Results for saccades and switching. **(A)** Patients with EOAD showed more frequent saccades than NC participants. **(B)** Patients with EOAD showed more frequent switching than patients with LOAD and NC participants did. EOAD, early-onset Alzheimer's disease; LOAD, late-onset Alzheimer's disease; NC, normal control. *p < 0.05, **p < 0.01, ***p < 0.001, ****p < 0.0001.

The median number of saccades was 267 in the EOAD group, 169 in the LOAD group, and 105 in the NC group, and those differences were significant (KW test, *p* < 0.001). The number of saccades was higher in patients with EOAD than in NC participants (Dunn's pairwise tests, *p* < 0.001, EOAD vs. NC) (Figure 4A). The median total duration of saccades was 4.34 s in EOAD, 3.99 s in LOAD, and 4.32 s in NC participants, and those differences among groups were not significant (KW test, *p* = 0.587).

#### Switches Between the Two AOIs

The median number of switches was 80 in patients with EOAD, 50 in patients with LOAD, and 36 in NC participants, which was a significant difference among groups (KW test, *p* < 0.001). The number of switches was higher in the EOAD group than in the LOAD and NC groups (Dunn's pairwise tests, EOAD vs. LOAD: *p* = 0. 049, EOAD vs. NC: *p* < 0.001) (Figure 4B).

### Correlation Between the Eye-Tracking Metrics and the Scores of Neuropsychological Tests

We compared the eye-tracking metrics with the scores of neuropsychological tests after data from EOAD and LOAD individuals were combined (Table 3).

**Table 3:** The correlation coefficient between eye-tracking metrics and neuropsychological test scores in the combined EOAD and LOAD groups.

	**Total number of fixation**	**Number of fixation (perceptual AOI)**	**Number of fixation (working AOI)**	**Ratio of number (working/** **perceptual AOI)**	**Total fixation duration**	**Fixation duration (perceptual AOI)**	**Fixation duration (working AOI)**	**Ratio of duration (working/** **perceptual AOI)**	**Total number of saccades**	**Total duration of saccades**	**Number of switches**
Forward digit span	−0.269	−0.246	−0.202	0.210	−0.259	−0.248	−0.152	0.207	−0.244	0.053	−0.175
Backward digit span	−0.288	−0.155	−0.383^*^	0.184	−0.299	−0.189	−0.332	0.228	−0.326	0.049	−0.199
K-BNT	−0.016	−0.044	0.032	0.240	−0.066	−0.111	0.026	0.190	−0.068	0.053	0.077
Calculation	−0.669^***^	−0.725^***^	−0.436^*^	0.431^*^	−0.658^***^	−0.749^***^	−0.340	0.500^**^	−0.668^***^	−0.051	−0.458^*^
SVLT: immediate recall	0.020	0.099	−0.110	0.107	−0.007	0.058	−0.087	0.164	−0.092	−0.106	−0.152
SVLT: delayed recall	0.500^**^	0.609^***^	0.138	−0.256	0.431^*^	0.535^**^	0.101	−0.265	0.301	−0.208	0.152
SVLT: recognition	0.038	0.166	−0.175	−0.034	0.034	0.145	−0.148	0.027	−0.065	−0.197	−0.117
RCFT: copying	−0.443^*^	−0.580^**^	−0.171	0.514^**^	−0.426^*^	−0.598^**^	−0.106	0.566^**^	−0.438^*^	−0.059	−0.215
RCFT: immediate recall	−0.161	−0.234	−0.036	0.173	−0.085	−0.206	0.083	0.137	−0.140	−0.165	−0.013
RCFT: delayed recall	−0.059	−0.094	−0.004	0.041	0.010	−0.065	0.101	0.027	−0.053	−0.235	0.018
RCFT: recognition	0.040	−0.101	0.242	0.372^*^	−0.014	−0.104	0.099	0.235	0.075	0.332	0.150
COWAT: animal	−0.291	−0.276	−0.204	0.408^*^	−0.342	−0.320	−0.233	0.365^*^	−0.359	0.000	−0.162
COWAT: supermarket	−0.322	−0.289	−0.250	0.382^*^	−0.349	−0.337	−0.224	0.432^*^	−0.387^*^	−0.021	−0.263
COWAT: phonemic	−0.417^*^	−0.365	−0.338	0.321	−0.395^*^	−0.368	−0.258	0.375^*^	−0.416^*^	−0.041	−0.300
Stroop test: color	−0.386^*^	−0.520^**^	−0.132	0.595^**^	−0.398^*^	−0.521^**^	−0.136	0.596^**^	−0.384^*^	0.056	−0.210
MMSE	−0.114	−0.008	−0.235	0.208	−0.148	−0.074	−0.190	0.258	−0.244	−0.112	−0.134

* *p < 0.05*.

** *p < 0.01*.

*** *p < 0.001*.

*AOI, area of interest; COWAT, Controlled Oral Word Association Test; K-BNT, Korean version of the Boston Naming Test; MMSE, Mini-Mental State Examination; RCFT, Rey–Osterrieth complex figure test; SVLT, Seoul Verbal Learning Test*.

The total number of fixations correlated negatively with calculation, RCFT copying, COWAT phonemic, and Stroop test scores. The number of fixations on the perceptual AOI also correlated negatively with calculation, RCFT copying, and Stroop test scores. The number of fixations on the working AOI negatively correlated with digit span backward and calculation scores. The ratio of the number of fixations between the two AOIs (working space/perceptual space) correlated positively with calculation, RCFT copying, RCFT recognition, COWAT animal and supermarket, and Stroop test scores.

The total fixations duration correlated negatively with calculation, RCFT copying, COWAT phonemic, and Stroop test scores. The fixation duration on the perceptual AOI correlated negatively with calculation, RCFT copying, and Stroop test scores. The number of fixations on the working AOI did not correlate with any of the scores. The ratio of the fixation duration between the two AOIs (working space/perceptual space) correlated positively with calculation, RCFT copying, COWAT (animal/supermarket /phonemic), and Stroop test scores.

The number of saccades correlated negatively with calculation, RCFT copying, COWAT (supermarket /phonemic), and Stroop test scores. The duration of saccades did not correlate with any of the scores. The number of switches correlated negatively with calculation.

## Discussion

In this study, we used eye-tracking glasses to assess eye-tracking metrics in patients with EOAD, patients with LOAD, and NC participants while they copied the simplified RCFT, which was created and validated in our previous study (39). We constructed perceptual and working AOIs to investigate the different stages of copying, and then, we compared the number and duration of fixations and saccades, and number of switches across the two AOIs, between the two patient groups with AD and the NC participants. In summary, the total number and duration of fixations showed an overall trend to decrease in the order of EOAD, LOAD, and NC participants. Statistical analyses showed that the total number of fixations was greater in the patients with EOAD than the NC participants; the total fixation duration of both patient groups with AD was longer in than that of the NC participants. On the perceptual AOI, the number and duration of fixations were greater in both patient groups with AD than in the NC participants. On the working AOI, the number and duration of fixations were greater in the patients with EOAD than in the patients with LOAD and NC participants. The number of saccades was greater in patients with EOAD than in NC participants. The number of switches was greater in the EOAD group than in the patients with LOAD and NC participants. The eye-tracking metrics from the simplified RCFT correlated with the neuropsychological test scores.

Several studies have shown that eye-tracking measurements could be useful in evaluating visual cognition (15–18). However, previous studies used simple targets, such as a cross or a circle, and asked participants to look at static (fixation) or moving (pursuit) targets or engage in saccade–antisaccade tasks, which might be insufficient for evaluating complex visuospatial and visuoconstructional functioning (15, 16, 18). In contrast, we created an experimental setting almost identical to a conventional neuropsychological test setting in which participants copy a complex figure. The only difference between our experimental setting and the standard neuropsychological test setting was that participants wore an eye tracker. We even used a lightweight (47 g) glasses-type eye tracker that did not restrict head movement or eye blinking to closely simulate a natural copying process.

Furthermore, we divided the copying process into three stages: looking closely at the sample figure to reflect visuoperceptual function; holding the information about the sample figure in the visuospatial sketchpad (12) to reflect working memory function, and drawing or constructing the figure seen in the perceptual space to reflect visuoconstructional function. Previous studies also employed the decomposition of copying processes into input (visuoperception) and output components that were labeled under different names such as visuoconstruction or graphic production (10, 11). Other studies highlighted aspects of working memory when drawing complex figures (13, 14). For instance, the closing-in phenomenon, a common behavior seen in patients with AD, might be a typical example that can be explained by a working memory hypothesis (42, 43). Briefly, patients with AD were requested to draw figures or lines that varied in the complexity and showed that the more complex the target figures were, the nearer the patients drew toward the target, which can be compensatory strategies to overcome visuospatial working memory deficits (42, 43).

Overall, there was a trend that our patients with AD showed a greater number and duration of fixations compared with NC participants, when the results on the perceptual and working AOIs were combined. The patients with AD also showed a longer fixation duration and greater number of fixations on the perceptual AOI than the NC participants. Multiple linear regression analyses adjusting for completion time further supported our results such that the ratio of number (working/perceptual AOI) and the ratio of duration (working/perceptual AOI) measurements were associated with both AD vs. NC group differences. Fixations are stationary points for the eyes and represent a visual intake of information. Therefore, an increased number of fixations and fixation duration on the perceptual AOI indicate an increase in target processing times. We also found that the number and duration of fixations on perceptual AOI showed a moderate to strong negative correlation with calculation, RCFT copying, and Stroop test scores. These results support that increased number and duration of fixations on perceptual AOI can represent impairment in frontoparietal function. Consistent with our results, previous studies have demonstrated that patients with AD show abnormal eye-tracking metrics, including fixations, during simple tasks (17, 44, 45). While visually searching for a target in a distracting environment, patients with AD had a notably greater number of fixations and longer fixation duration than the NC participants (44, 45). On the other hand, when NC participants and patients with AD are asked only to look at a target picture without the pressure of remembering or copying the figure, the fixation metric results vary from study to study (46). Therefore, taking previous studies and our study together, the results indicated that a greater number and duration of fixations on perceptual AOI are required while visually encoding complex figures, a neurobehavior that might be related to visuoperceptual dysfunctions in patients with AD. Regarding the mechanism of longer fixations in our patients with AD compared to controls, we simply attributed it to the assumption that patients with AD spend more time encoding the visual information. However, we cannot exclude the possibility that patients with AD with parietal and frontal injuries lost their inhibitory control for salient stimuli; therefore, once they fixate their gaze on certain stimuli, they may have difficulty disengaging from those stimuli, resulting in longer fixations.

Unlike the fixations on the perceptual AOI, where the two AD groups were comparable, the EOAD group showed a greater number of fixations and longer fixation duration on the working AOI compared with the patients with LOAD. Multiple linear regression after adjusting for the completion time of drawing also supported the EOAD vs. LOAD group difference in terms of number and duration of fixations on the working AOI. These results suggest that patients with EOAD are more impaired in their visuoconstructional functioning, as assessed by number of fixations and fixation duration on the working AOI, than patients with LOAD because the two patient groups with AD had the same level of visuoperceptual deficits as assessed by number of fixations and duration on the perceptual AOI. Our results support a previous study that showed severe visuoconstructional dysfunction in the patients with EOAD than LOAD, whereas visuospatial functioning, probably visuoperceptual functioning, did not differ significantly between them (7). Why patients with EOAD have more severe visuoconstructional dysfunction (as assessed by the number of fixations and fixation duration on the working AOI) than patients with LOAD remains unclear. However, visuoconstructional tasks require strategies for planning, structuring, and coordination. Therefore, we speculate that the executive functions that are responsible for those strategies were more impaired in patients with EOAD than in LOAD (7). Alternative account might be that it has been known that frontal involvement is more severe in patients with EOAD than LOAD (47) when they have reached the same level of disease severity, therefore, motor perseveration of hand and eye movements associated with frontal injury might have contributed to the longer fixations in the working space.

Patients with EOAD, not LOAD, showed more frequent saccades than NC participants. Saccades are rapid eye movements that occur between fixations. Various saccadic dysfunctions have been reported in patients with AD (46), but previous studies have focused on the performance of antisaccade tasks in a restricted experimental design (18). In contrast, our participants were not asked to perform pro- or antisaccade tasks; they were instead exposed to an everyday experimental setting of drawing a picture. According to the Posner paradigm, visual attention is divided into three steps: disengagement of current focus, which is known to be associated with the frontoparietal network; movement to the selected target; and engagement with the selected target, which is also known to be associated with the frontoparietal network (48). The frontoparietal network is thus a partly overlapping circuit for both the disengagement and engagement steps. However, the disengagement step is more associated with the dorsal frontoparietal network (including the frontal eye field, the superior parietal lobule, and the intraparietal sulcus), and the engagement step is more associated with the ventral frontoparietal network (including the frontal eye field, the middle and inferior frontal gyri, and the temporoparietal junction). It is well known that temporoparietal atrophy is a marker of AD pathology, and FDG-PET (47) and MRI studies (49, 50) have shown that patients with EOAD have more parietal injury than patients with LOAD. The posterior parietal cortex is essential in controlling both eye movements and attention (51). Therefore, patients with AD, especially patients with EOAD, might have shown more frequent saccades as a result of bilateral parietal dysfunction.

Patients with EOAD also switched between the two AOIs more frequently than the patients with LOAD and NC participants. Switches are saccades between the perceptual space (blue rectangle) and the working space (red rectangle) (Figure 1B). More frequent switching between the two figures could indicate that patients with EOAD have more severe working memory deficits than patients with LOAD and NC participants. To copy a figure, one has to remember the target figure (working memory) from the perceptual AOI for a while, but if the working memory is impaired, the participant would need to study the figure repeatedly.

The strengths of our study are that, to our knowledge, this is the first paper to separately investigate eye-tracking behaviors in perceptual and working spaces while participants draw a complex figure in a user-friendly environment or natural drawing setting that could happen in daily life. Second, we assessed not only the final copying score of each completed figure but also subjects' eye behaviors during the process of drawing. Among eye-tracking metrics, number and duration of fixations on the perceptual space AOI discriminated patients with EOAD or LOAD from NC participants. These eye-tracking metrics might be more sensitive than the final copying scores in detecting early signs of AD. Moreover, increased number and duration of fixations on the working space AOI and number of switches can help to distinguish patients with EOAD from LOAD. Patients with EOAD and LOAD achieved comparable levels of performance with simplified RCFT and original RCFT scores. However, Patients with EOAD showed greater number and duration of fixations on the working space AOI and more frequent switching between AOIs, which might reflect more mental efforts to achieve the similar level of performance. In that way, our study broadens the understanding of the visuoperceptual and visuoconstructional impairment patterns of patients with EOAD and LOAD. However, our study also has the limitation that although RCFT performances involve multiple domains of cognitive and neural processes from basic motor and visual functions to visuomotor coordination and attention/executive function in addition to higher levels of visuospatial function, we simplified this complicated process into three components. To minimize the effect of basic visual and motor functions, we excluded participants with visual and motor problems from this study. Nevertheless, the effects of factors we did not address in this study should be investigated in the future studies. Also, our data did not support our hypothesis that patients with EOAD and LOAD would differ in terms of total number and duration of fixation and number of saccade, which might be due to the relatively small sample size. Therefore, future standardization studies with a large number of healthy participants are warranted. In addition, we need to compare eye-tracking metrics during the copying process in patients with a different disease severity and between amyloid positive and negative patients.

## Data Availability Statement

The raw data supporting the conclusions of this article will be made available by the authors, without undue reservation.

## Ethics Statement

The studies involving human participants were reviewed and approved by Institutional Review Board of Samsung Medical Center. The patients/participants provided their written informed consent to participate in this study.

## Author Contributions

DN, JChi, and BL contributed to conception and design of the study. KK performed the statistical analysis and wrote the first draft of the manuscript. JCho organized the database and wrote sections of the manuscript. All authors contributed to manuscript revision, read, and approved the submitted version.

## Funding

This work was supported by a National Research Foundation of Korea (NRF) grant funded by the Korean government (MSIT) NRF 2020R1G1A1102644, the National Research Council of Science and Technology (NST) grant by the Korea government (MSIP) (No. CRC15 04 KIST), and the Fund of Biomedical Research Institute, Jeonbuk National University Hospital.

## Conflict of Interest

The authors declare that the research was conducted in the absence of any commercial or financial relationships that could be construed as a potential conflict of interest.

## Publisher's Note

All claims expressed in this article are solely those of the authors and do not necessarily represent those of their affiliated organizations, or those of the publisher, the editors and the reviewers. Any product that may be evaluated in this article, or claim that may be made by its manufacturer, is not guaranteed or endorsed by the publisher.

## Abbreviations

AD: Alzheimer's disease
AOIs: Areas of interest
CDR: Clinical Dementia Rating
COWAT: Controlled Oral Word Association Test
EOAD: Early-onset Alzheimer's disease
K-BNT: Korean version of the Boston naming test
LOAD: Late-onset Alzheimer's disease
MMSE: Mini-Mental State Examination
MRI: Magnetic resonance imaging
NC: Normal control
RCFT: Rey–Osterrieth complex figure test
SNSB: Seoul Neuropsychological Screening Battery
SVLT: Seoul Verbal Learning Test.
